# Glucosinolates Extracts from *Brassica juncea* Ameliorate HFD-Induced Non-Alcoholic Steatohepatitis

**DOI:** 10.3390/nu15163497

**Published:** 2023-08-08

**Authors:** Ming-Jen Sheu, Mei-Chen Yeh, Ming-Chang Tsai, Chi-Chih Wang, Yen-Ling Chang, Chau-Jong Wang, Hui-Pei Huang

**Affiliations:** 1 Division of Hepatogastroenterology, Department of Internal Medicine, Chi Mei Medical Center, No. 901, Zhonghua Rd. Yongkang Dist., Tainan City 71004, Taiwan; hmj@mail.chimei.org.tw; 2 Division of Metabolism and Endocrinology, Department of Internal Medicine, Chi Mei Medical Center, Tainan 71004, Taiwan; jamie.wien@gmail.com; 3 Institute of Medicine, Chung Shan Medical University, Taichung 402, Taiwan; tsaimc1110@gmail.com (M.-C.T.); bananaudwang@gmail.com (C.-C.W.); hhpei0422@gmail.com (Y.-L.C.); 4 School of Medicine, Chung Shan Medical University, Taichung 402, Taiwan; 5 Division of Gastroenterology and Hepatology, Department of Internal Medicine, Chung Shan Medical University Hospital, Taichung 402, Taiwan; 6 Department of Health Industry Technology Management, Chung Shan Medical University, Taichung 402, Taiwan; 7 Department of Medical Research, Chung Shan Medical University Hospital, Taichung 402, Taiwan; 8 Department of Biochemistry, School of Medicine, Chung Shan Medical University, Taichung 40242, Taiwan

**Keywords:** non-alcoholic fatty liver disease (NAFLD), *Brassica juncea*, glucosinolates, high-fat diet (HFD), AMPK

## Abstract

Non-alcoholic fatty liver disease (NAFLD) is mainly characterized by excessive fat accumulation in the liver. It spans a spectrum of diseases from hepatic steatosis to non-alcoholic steatohepatitis (NASH), fibrosis, cirrhosis, and hepatocellular carcinoma (HCC). *Brassica juncea* is rich in glucosinolates and has been proven to possess many potential pharmacological properties, including hypoglycemic, anti-oxidation, anti-inflammatory, and anti-carcinogenic activities. This study aims to investigate whether whole-plant *Brassica juncea* (WBJ) and its glucosinolates extracts (BGE) have hepatoprotective effects against a high-fat diet (HFD)-induced NAFLD and further explore the mechanism underlying this process in vivo and in vitro. WBJ treatment significantly reduced body fat, dyslipidemia, hepatic steatosis, liver injury, and inflammation; WBJ treatment also reversed the antioxidant enzyme activity to attenuate oxidative stress in HFD-fed rat liver. Moreover, WBJ and BGE enhanced the activation of AMPK to reduce SREBPs, fatty acid synthase, and HMG-CoA reductase but increased the expression of CPT-I and PPARα to improve hepatic steatosis. In addition, WBJ and BGE could ameliorate NAFLD by inhibiting TNF-α and NF-κB. Based on the above results, this study demonstrates that WBJ and BGE ameliorate HFD-induced hepatic steatosis and liver injury. Therefore, these treatments could represent an unprecedented hope toward improved strategies for NAFLD.

## 1. Introduction

Worldwide, the incidence of chronic liver disease is increasing year by year, resulting in liver-related morbidity and increased mortality [1]. Non-alcoholic fatty liver disease (NAFLD) is the most common causes of chronic liver disease. Many studies have clearly shown that NAFLD is a multi-organ disease that is related to type 2 diabetes mellitus (T2DM), cardiovascular diseases (CVD), and chronic kidney disease (CKD) [2]. Methods to effectively ameliorate NAFLD have become a global issue that should not be underestimated. NAFLD is related to lipotoxicity, which is caused by the accumulation of toxic metabolites derived from triglycerides in the liver, pancreas, and muscles, leading to a cascade of inflammation and insulin resistance [3]. The pathogenic mechanism of NAFLD is still unclear. So far, the two-hit theory proposed by Day et al. is the most widely accepted hypothesis [4]. The first hit is brought about by obesity and insulin resistance (IR). IR can promote the synthesis of triglycerides and reduce fatty acid β-oxidation in mitochondria, which accelerates the accumulation of excessive triglycerides and free fatty acids in the liver, resulting in hepatic steatosis [5]. The second hit is related to oxidative stress and abnormal mitochondrial function. Severe steatosis leads to lipid toxicity, induces the increase in the CYP2E1 and CYP4A activities of cytochrome P-450, produces oxidative free radicals, and releases inflammatory cytokines (e.g., TNF-α, IL-6, etc.), which eventually causes more serious complications such as steatohepatitis, liver fibrosis, liver cirrhosis, or liver cancer [6].

Research has demonstrated that, if dysregulated, AMPK and its related pathways are associated with metabolic diseases such as cancer, inflammatory diseases, obesity, and diabetes. AMPK activates the upstream kinases of 3-hydroxy-3-methylglutaryl-CoA reductase (HMG CoAR) and acetyl-CoA carboxylase (ACC), which, in turn, phosphorylate HMG CoAR and ACC, therefore inhibiting cholesterol biosynthesis and fatty acid synthesis/lipogenesis [7]. In addition, AMPK inhibits glycolysis to control hepatic glucose production by downregulating phosphoenolpyruvate carboxykinase and glucose 6-phosphatase [8]. Therefore, restoring or activating the expression of the AMPK pathway could potentially be therapeutic in NAFLD.

Peroxisome proliferator-activated receptor α (PPARα) is a ligand-activated transcription factor that is essential for fatty acid metabolism [9]. Many studies have shown that fatty acids are lipophilic molecules, which can be used as ligands to activate PPARα. Activated PPARα triggers several genes, including lipoprotein lipase genes that allow lipoprotein particles to release fatty acids, which conversely control the rates of fat catabolism, lipogenesis, and ketone body synthesis [10]. Therefore, PPARα is expressed in many organs with active fatty acid oxidation such as the liver, heart, and skeletal muscle [11]. The influences of PPARα on both acute and chronic inflammatory processes have been identified in in vitro and in vivo studies. PPARα are essential to the prevention of NAFLD, and extra-hepatocyte PPARα activity contributes to whole-body lipid homeostasis. Moreover, PPARα also improves metabolic syndromes, including obesity, atherosclerosis, and NASH, by inhibiting chronic inflammation [12]. For instance, PPARα negatively regulates hepatic inflammatory responses induced by an improper diet by repressing the signaling pathways controlled by the AP-1 and NF-κB transcription factors [13].

*Brassica juncea* (mustard) contains ascorbic acid, carotenoids, fiber, polyphenols, glucosinolates, and other ingredients, which have the effects of improving metabolic syndromes such as obesity, dyslipidemia, and diabetes [14]. Polyphenols and glucosinolates are considered to be the main functional components for disease prevention. Many studies have confirmed that glucosinolates have the effects of anti-inflammation and anti-oxidation, increasing the activity of detoxifying enzymes and inhibiting the mitosis of cancer cells, thereby preventing the occurrence of cancer [15]. Glucosinolates and their hydrolysates can activate phase II detoxification enzymes and antioxidant enzymes, as well as eliminate reactive oxygen species and lipid peroxidation by regulating the Nrf2/ARE signaling pathway [16]. However, the potential effects of glucosinolates and the mechanism of their action against a high-fat diet (HFD)-induced NAFLD remain unclear. In this study, the efficacy and mechanism of *Brassica juncea* and its functional component, glucosinolates, were analyzed in improving NAFLD in vivo and in vitro.

## 2. Materials and Methods

### 2.1. Preparation of Whole-Plant Brassica juncea (WBJ) and Glucosinolates Extracts from Brassica juncea (BGE)

Whole plants of *Brassica juncea* (WBJ) were freeze-dried using a rotary evaporator. The glucosinolates were extracted from 2 g WBJ with 20 mL 70% aqueous methanol solution in a water bath at 75 °C for 15 min. The extract was centrifuged at 3000 rpm for 5 min, and the supernatant was collected. All of the supernatant was pipetted onto a column containing a DEAE Sephadex A-25 anion exchange resin (GE Healthcare, Uppsala, Sweden). Glucosinolates were eluted from the column with 0.5 mol/L potassium sulphate. The eluent was vacuum freeze-dried to yield a powder as the glucosinolates extract of *Brassica juncea* (BGE). All BGE were dissolved with sterile ddH_2_O and filtered through a 0.22 μm syringe filter and stored at −20 °C until analysis with high-performance liquid chromatography (HPLC) or until performance of the cell experiment.

### 2.2. HPLC Analysis

The glucosinolate composition in *Brassica Juncea* was determined using HPLC according to a previous study [17]. The HPLC system (Hitachi, Danbury, CT, USA) consisted of a pump (L-6200A), an ultraviolet detector (L-4250), and a Hitachi D-7000 HPLC system manager program. Then, 10 μL BGE was injected into the GL Sciences Inertsil ODS-2 column (5 μm particle size, 4.6 × 250 mm). Mobile-phase solvent A (100% distilled water) and solvent B (20% acetonitrile in water) were used for the elution of compounds at a flow rate of 1.5 mL/min. The absorbance spectrum was detected at a wavelength of 229 nm. Sinigrin (SIN), progoitrin (PRO), and gluconapin (NAP) were used as standards.

### 2.3. Animal Models and Treatment

**Figure 1:**
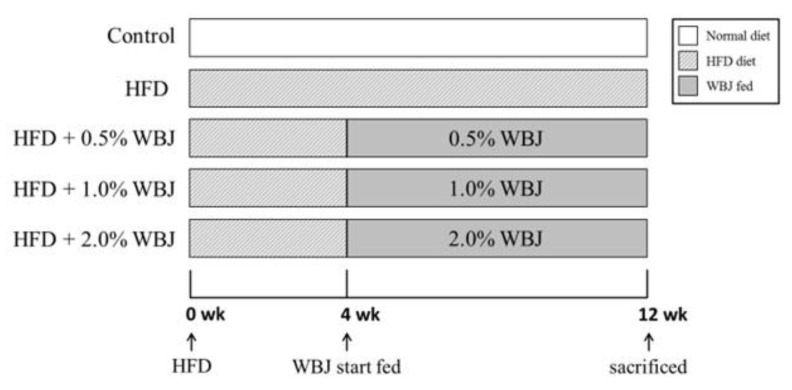
Schematic representation of WBJ treatment in Wistar rats.

Male Wistar rats (200 ± 10 g) were purchased from the BioLASCO Taiwan Co., Ltd. (Taipei, Taiwan). The rats were housed and used according to the guidelines of the Taiwan regulations for animal care. All animal experimental protocols were reviewed and approved by the Institutional Animal Care and Use Committee (IACUC) of the Chung Shan Medical University, Taichung, Taiwan (Approval Number 1770). The rats were housed in standard laboratory conditions (22 ± 2 °C, 60 ± 5% relative humidity, and under a 12 h light/dark cycle). Food and water were provided ad libitum to help rats adapt to the new environment for at least 1 week before the experimental procedures. In total, 60 rats were randomly divided into 5 groups (n = 12 per group): control; HFD; HFD + 0.5% WBJ; HFD + 1.0% WBJ; HFD + 2.0% WBJ (Figure 1). The feed formulation for the rats is presented in Table 1. All rats except the control group were fed with the HFD for 4 weeks prior to WBJ administration to trigger non-alcoholic steatohepatitis. After 4 weeks, the rats were fed an HFD and given different concentrations of WBJ (0.5%, 1.0%, and 2.0%) simultaneously for 8 weeks. During this period, all rats were weighed every week. At the end of the experiment, all animals were euthanized, and the liver, blood, and visceral fat were subsequently harvested for further analysis.

### 2.4. Blood Sample Analysis

Samples were harvested with cardiac puncture and immediately collected into BD vacutainer TM heparin blood collection tubes. The blood samples were centrifuged at 3000 rpm for 5 min at 4 °C. The serum was separated and stored at −20 °C for further analysis. The levels of total cholesterol, triglyceride, aspartate transaminase (AST), and alanine transaminase (ALT) in the serum were measured with clinical chemistry reagent kits (Randox Laboratories, Ltd., Antrim, UK). The free fatty acid (FFA) level in the serum was determined with a quantification kit (BioVision, Inc., Mountain View, CA, USA) according to the manufacturer’s instructions.

### 2.5. Hepatic Histologic Analysis

Liver tissues were excised and fixed in 10% (*v/v*) neutral buffered formalin overnight. Then, the specimens were paraffin embedded. The paraffin blocks were sectioned at 5 μm and stained with a hematoxylin and eosin (H&E) reagent. Frozen sections were stained with Oil red O. The sections were observed at 200× or 400× magnification under optical microscopy.

### 2.6. Determination of Total Triglyceride and Cholesterol Contents in the Liver

The rat liver samples (0.5 g) were homogenized with chloroform/methanol (1:2, *v*/*v*) at 4 °C for 3 min. Then, chloroform and distilled water (1:1, *v*/*v*) were added to the homogenate and vortexed for 1 min. The homogenate was centrifuged at 3000 rpm for 12 min, and the lipid extract in the lowest layer was dissolved with 200 μL isopropanol after it was completely air dried. The triglyceride and cholesterol levels in the hepatic samples were measured through commercial kits (Human, Wiesbaden, Germany) according to the manufacturer’s protocol.

### 2.7. Antioxidant Enzyme Activity Assays

The rat liver samples (0.5 g) were mechanically disrupted with 5 mL phosphate–EDTA (0.1 M KH_2_PO_4_ + 0.1 mM EDTA, pH 7.0) buffer with a homogenizer at 4 °C for 3 min. The homogenate was centrifuged at 3000 rpm for 30 min. After that, the supernatant was recentrifuged at 12,000 rpm for 5 min. The cleared hepatic homogenate was collected and stored at −20 °C. The activity of SOD was detected according to a previous method [18]. Then, 50 μL hepatic homogenate was mixed with 100 μL Tris–cacodylic acid buffer (50 mM, pH 8.2) and ultrapure water added to 980 μL. Lastly, 20 μL 0.2 mM pyrogallol was blended in the mixed solution. The autoxidation rate of pyrogallol was immediately assayed every 30 s for 3 min at 420 nm with a spectrophotometer and calculated to analyze the superoxide anion radical scavenging activity of SOD. The glutathione (GSH) activity was measured as follows: 0.5 mL hepatic homogenate was mixed with 4.5 mL phosphate–EDTA buffer (0.1 M Na_2_HPO_4_, 5 mM EDTA, pH 8.0), and the mixture was diluted 10 times in phosphate–EDTA buffer. After diluting, 0.1 mL o-phthalaldehyde (5 mg/mL, dissolved in ethanol) was mixed with the dilute solution. The final reaction was performed for 15 min at room temperature and protected from light. The fluorescence intensity was detected at an emission spectrum of 420 nm and an excitation spectrum of 350 nm through a fluorophotometer. GSH-Rd activity was assayed as described previously [19]. Then, 10 μL hepatic homogenate was mixed with 90 μL potassium phosphate buffer solution (20 mM, pH 7.0), followed by incubation in 900 μL of the reaction solution (1.1 mM MgCl_2_•6H_2_O, 5.0 mM GSSG and 0.1 mM NADPH in potassium phosphate buffer (100 mM, pH7.0)). The reduction rate of NADPH was immediately monitored every 30 s for 5 min at a wavelength of 340 nm with a spectrophotometer.

### 2.8. Cell Culture and Treatment

The human hepatocellular carcinoma cell line HepG2 was purchased as a commercially available product (Food Industry Research and Development Institute, Hsinchu, Taiwan). HepG2 cells were cultured in high-glucose Dulbecco’s Modified Eagle Medium (DMEM) that was supplemented with 10% fetal bovine serum (FBS), 1.5 g/L sodium bicarbonate, 4 mM L-glutamine, 1 mM sodium pyruvate, 1 mM non-essential amino acids solution (NEAA), and 1 mM PSA. The cultured cells were grown at 37 °C in a humidified incubator with 5% CO_2_. To establish the in vitro model of cellular fat accumulation, HepG2 cells at 70% confluence were stimulated with 0.3 mM of a long-chain oleic acid (OA)/1% BSA complex and treated with various concentrations of BGE (50 μM SIN, 50 μM PRO, 50 μM NAP, and 2.0–6.0 mg/mL BGE) for 24 h.

### 2.9. Cell Viability and Cytotoxicity Assays

Cell viability was calculated through the reduction of MTT (3-(4,5-dimethylthiazol-2-yl)-2,5-diphenyltetrazolium bromide). Cells were frequently cultured for 24 h, followed by treatment with 0.3 mM OA/1% BSA and various concentrations of BGE for 24 h. Thereafter, the medium was removed, and the MTT reagent (0.5 mg/mL in culture medium) was subsequently added to each well for 4 h. The reduced purple-blue MTT formazan crystals produced by viable cells were solubilized with 1 mL isopropanol, and the solution was centrifuged at 1200 rpm for 5 min. After centrifuging, the supernatant was collected and measured at 563 nm with an ELISA reader.

### 2.10. Nile Red Stain

Cellular lipid accumulation was assessed with Nile red staining. HepG2 cells were treated with 0.3 mM OA/1% BSA and various concentrations of BGE (50 μM SIN, 50 μM PRO, 50 μM NAP, and 2.0 mg/mL BGE) for 24 h. Adherent cells were separated with trypsin for 5 min and then centrifuged at 1000 rpm for 10 min. The supernatant was removed and washed twice with PBS. Cells stained with the Nile red solution were removed from light exposure for 30 min at room temperature. The cellular lipid contents were analyzed with flow cytometry (BD Biosciences, Franklin Lakes, NJ, USA).

### 2.11. Preparation of Cells and Hepatic Proteins

The protein extracts from cultured cells or liver tissues were lysed and extracted with a RIPA buffer (150 mM NaCl, 0.5% deoxycholic acid, 50 mM Tris-Base, 1% NP-40, 1% SDS, 10 μg/mL PMSF, and 10 μg/mL leupeptin, pH 7.5) containing 1% protease inhibitor (17 μg/mL leupeptin and 10 μg/mL sodium orthovanadate) and phosphatase inhibitor. The cell lysates or liver tissues were homogenized with the RIPA buffer on ice for 3 min. Then, all protein extracts were centrifuged at 12,000 rpm for 20 min at 4 °C. The protein concentration was measured with Coomassie blue (Kenlor Industries, Inc., Santa Ana, CA, USA) and BSA (bovine serum albumin) was used as a standard. The absorbance was detected at 595 nm with a spectrophotometer.

### 2.12. Western Blot Analysis

Protein samples were separated with a 6–15% SDS-PAGE and transferred onto nitrocellulose membranes. Membranes were blocked with commercial BlockPRO Protein-Free blocking buffer (Visual Protein) for 1 h at room temperature. Blots were washed with TBST (Tris-buffered saline with Tween 20, pH7.6) 3 times (10 min each time) and hybridized with various primary antibodies specific for p-AMPK, AMPK, SREBP-1, FAS, SREBP-2, HMGCoR, CPT1, TNF-α, or NF-κB overnight at 4 °C. The primary antibodies were removed, and the membranes were washed with TBST followed by incubation with anti-mouse or anti-rabbit horseradish peroxidase HRP-conjugated secondary antibodies for 1 h at room temperature. The secondary antibodies were removed, and the membranes were washed with TBST. The blotted membrane was activated with enhanced chemiluminescence (ECL) and imaged with a LAS-4000 Super CCD Remote Control Science Imaging System (Fuji, Tokyo, Japan). Protein quantity was determined with densitometry using Fujfilm MultiGauge, version 3.0, software.

### 2.13. Statistical Analysis

Statistical analyses were performed using SigmaPlot 12.5 software (12.5.0.38). The statistical significance (*p* < 0.05) among all of the different groups was determined using the student’s t test or one-way ANOVA. Results are expressed as the mean value ± SD. All results are representative of at least three independent experiments.

## 3. Results

### 3.1. Daily Intake of BGE in Rats

**Figure 2:**
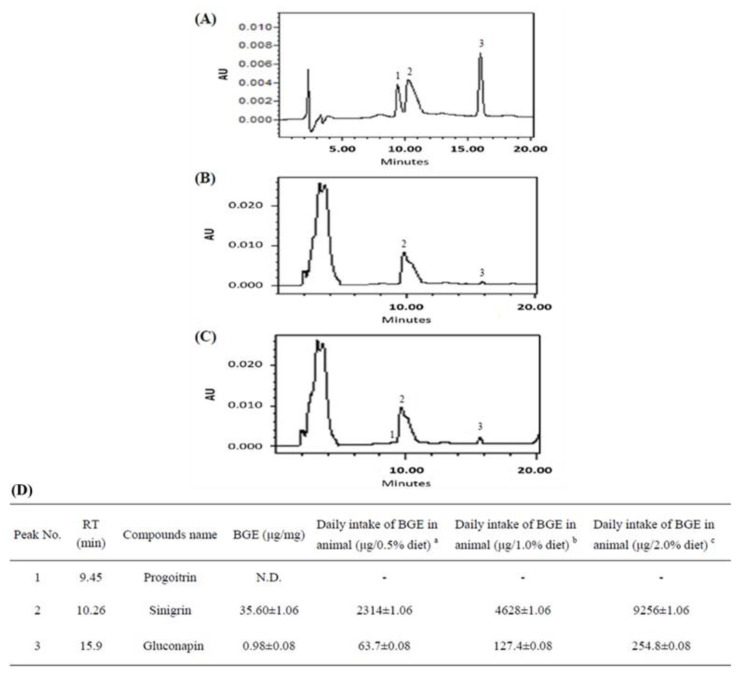
The HPLC chromatogram of BGE. (**A**) HPLC chromatogram of three kinds of glucosinolates standards. Peak: 1, sinigrin (SIN); 2, progoitrin (PRO); 3, gluconapin (NAP). (**B**) HPLC chromatogram of glucosinolates from BGE (100 mg/mL, 10 μL). (**C**) HPLC chromatogram profiles of mixture BGE and 2 μg/mL glucosinolate standards. (**D**) Glucosinolate compounds identified in BGE. ^a^ The average daily intake in the rats was 13 g/day and BGE was 0.5% of the diet. ^b^ The average daily intake in the rats was 13 g/day and BGE was 1.0% of the diet. ^c^ The average daily intake in the rats was 13 g/day and BGE was 2.0% of the diet.

The major glucosinolates in BGE were sinigrin (SIN, 97.33%) and gluconapin (NAP, 2.67%) (Figure 2A–C). To simulate the pattern of human ingestion of *Brassica juncea*, the rats were directly fed whole-plant *Brassica juncea* powder (WBJ) to investigate the efficacy of *Brassica juncea* in NAFLD. According to the HPLC analysis, the daily food intake of rats of 0.5% WBJ, 1.0% WBJ, and 2.0% WBJ contained 2.314 g, 4.628 g, and 9.256 g of sinigrin and 63.7 μg, 127.4 μg, and 254.8 μg of gluconapin, respectively (Figure 2D).

### 3.2. Effect of WBJ Treatment on Serum Lipid Parameters

In order to reflect a human dietary pattern, the WBJ was freeze-dried and fed to obese rats induced by the HFD. After treatment with an HFD in rats, the serum triglyceride (TG) and free fatty acids (FFA) in the HFD group were higher than the control group, whereas WBJ decreased the TG level induced by the HFD. Low-density lipoprotein cholesterol (LDL-C) and high-density lipoprotein cholesterol (HDL-C) are clinically regarded as the key factors for evaluating cardiovascular disease [20]. This study further analyzed the contents of LDL-C and HDL-C in rat serum to explore whether WBJ could reduce blood lipids and reduce the incidence of cardiovascular disease. Table 2 shows that WBJ could decrease the total cholesterol content even though the HFD did not induce the increase in cholesterol in rats. The LDL-C level of the HFD group was higher than the control group. However, the value of the LDL-C after treatment with WBJ was lower than the HFD group. Meanwhile, after induction of the high-fat diet, the HDL-C in the HFD group was decreased. The value of HDL-C tended to rise in the WBJ treatment groups. A similar result was also observed for the LDL-C/HDL-C ratio.

The liver function in rat serum was also analyzed to further observe whether WBJ could reduce liver injury. The results showed that the GOT and GPT of the HFD group were higher than in the control group. After feeding the rats WBJ, the values decreased with the increase in WBJ concentration, which indicated that WBJ could alleviate the liver injury induced by the high-fat diet. However, WBJ had a less significant effect on improving blood sugar levels and renal function in this animal model (Table 2).

### 3.3. Effect of WBJ on Fat Distribution in Rats

**Figure 3:**
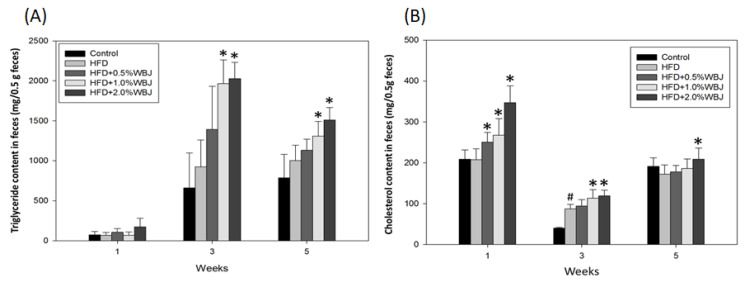
Effects of WBJ on lipid content in feces from HFD-fed rats. Feces levels of TG (**A**) and CHO (**B**) were measured at weeks 1, 3, and 5. Each value is expressed as the mean ± SD (n = 12/group). Results were statistically analyzed with one-way ANOVA. ^#^ *p* < 0.05 compared to the control group. * *p* < 0.05 compared to the HFD group.

The peripheral fat around the kidneys, intestinal interstitium, and accessory testicles were collected and weighed when the mice were euthanized. The results show that the fat around these organs in the group fed with the HFD increased significantly compared with the control group. The fat around the organs decreased when treated with WBJ, especially in the 2.0% WBJ group, indicating that WBJ could effectively reduce body fat production (Table 3). Similarly, the levels of TG and CHO in the feces of the HFD group were higher than in the control group, whereas the levels of TG and CHO in the feces increased with the increase in WBJ concentration compared to the HFD group (Figure 3). The above results reveal the effect of WBJ on the inhibition of lipid accumulation.

### 3.4. WBJ Reduced Hepatic Steatosis Induced by an HFD

**Figure 4:**
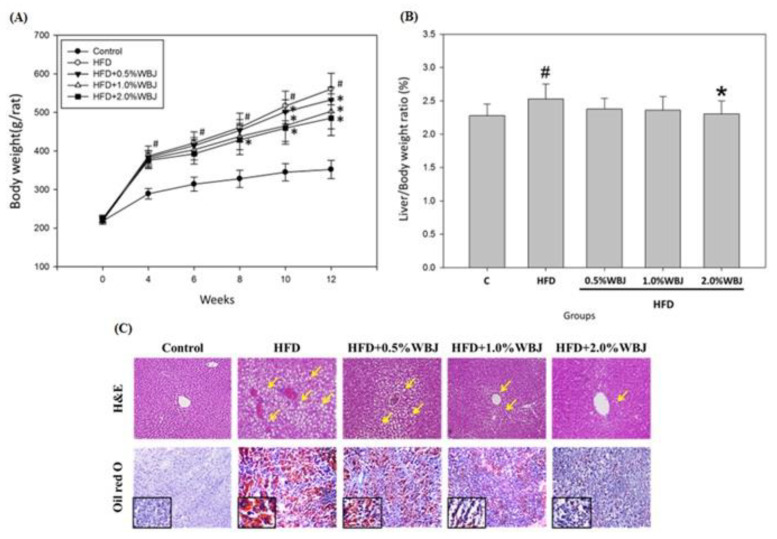
Effects of WBJ on the liver weight and histopathological examination of the liver tissue in HFD-fed rats. (**A**) Body weight change versus time in rats fed various diets. (**B**) Liver/body weight ratio. (**C**) H&E and Oil red O staining images of liver sections. All values are the mean ± SD (n = 12). Control, rats fed normal chow; HFD, rats fed with 40% beef tallow; HFD + 0.5% WBJ, rats fed an HFD with 0.5% WBJ; HFD + 1.0% WBJ, rats fed an HFD with 1.0% WBJ; HFD + 2.0% WBJ, rats were fed an HFD with 2.0% WBJ. Results were statistically analyzed with a one-way ANOVA. ^#^ *p* < 0.05 compared to the control group. * *p* < 0.05 compared to the HFD group. The sections were photographed at 200× or 400× magnification. The yellow arrow indicated the lipid droplet.

The liver weight/body weight ratio was used to evaluate whether WBJ ameliorated NAFLD induced by the high-fat diet. Compared with the HFD group, the ratio of liver weight to body weight was slightly decreased after feeding with WBJ. Notably, the ratio of liver weight to body weight in the 2.0% WBJ group was significantly lower than that in the HFD group (Figure 4A,B). Accordingly, WBJ could slow down hepatomegaly caused by a high-fat diet. Next, we observed the fat accumulation in rat livers with H&E staining and Oil red O staining. The results showed that there was no hepatic steatosis in the control group. However, many lipid droplets were found in the livers of the HFD group. The results indicate that WBJ could effectively reduce hepatic steatosis, especially in the 2.0% WBJ treatment group (Figure 4C).

### 3.5. Mechanisms of WBJ Reducing Hepatic Steatosis Induced by an HFD

**Figure 5:**
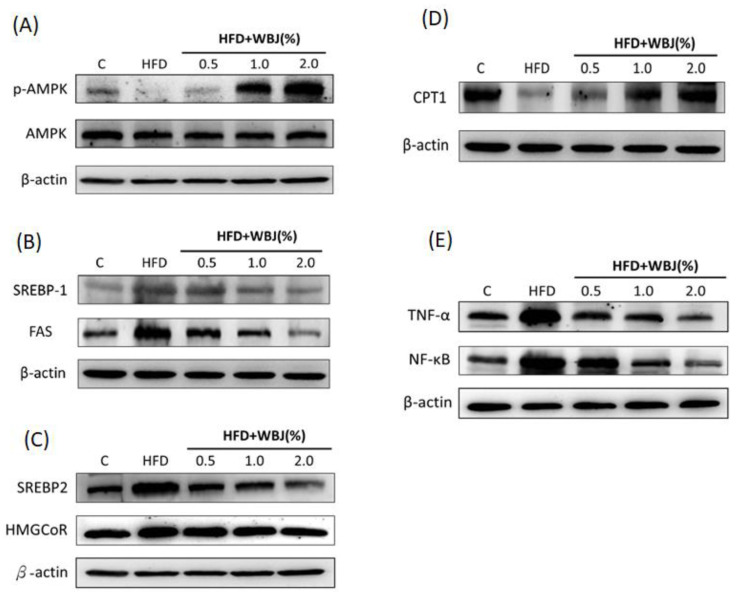
WBJ treatment reduced lipid synthesis-related protein and inflammatory protein expression in the liver of HFD-fed rats. Animals were fed a control diet, HFD, or HFD pair feeding with a 0.5%, 1.0%, or 2.0% WBJ diet (n = 12). Protein extracts from rat liver were measured with western blotting to detect (**A**) p-AMPK, AMPK; (**B**) SREBP-1, FAS; (**C**) SREBP-2, HMGCoR; (**D**) CPT1; and (**E**) TNF-α, NF-κb. β-actin was used as a loading control. Data are presented as the mean ± SD from three independent experiments and were statistically analyzed with a one-way ANOVA.

Our previous studies demonstrated that *Mulberry* leaf polyphenol extracts and *Solanum* polyphenol extracts reduced the expression of triglyceride synthesis (SREBP-1, FAS) and cholesterol synthesis (SREBP-2, HMGoCR), and increased the expression of the fatty acid β-oxidation protein CPT-1 by activating the AMPK pathway [21,22,23]. This study further observed the expression of lipid metabolism-related proteins with a western blot assay. The results showed that the activity of p-AMPK and the level of CPT-1 in the HFD group were lower than in the control group, and the expression of SREBP-1, FAS, SREBP-2, and HMGCoR was increased significantly. However, after treatment with WBJ, p-AMPK activity significantly increased compared to the HFD group. WBJ inhibited the expression of SREBP-1, FAS, SREBP-2, and HMGCoR induced by the HFD, and promoted CPT-1 expression (Figure 5A–D). Inflammation plays a crucial role in the development of NAFLD into non-alcoholic steatohepatitis (NASH). This study further analyzed the protein expression of inflammatory factors. The results showed that WBJ could reduce the activities of TNF-α and NF-κB in the liver induced by the HFD and had a significant effect at a low concentration of 0.5% WBJ (Figure 5E). In summary, it might be concluded that WBJ can produce lipid-lowering and anti-inflammatory effects by inhibiting the synthesis pathways of lipids and the expression of inflammatory factors, thereby reducing the occurrence of NAFLD.

### 3.6. WBJ Enhanced Antioxidative Enzyme Activities in Rat Liver

**Figure 6:**
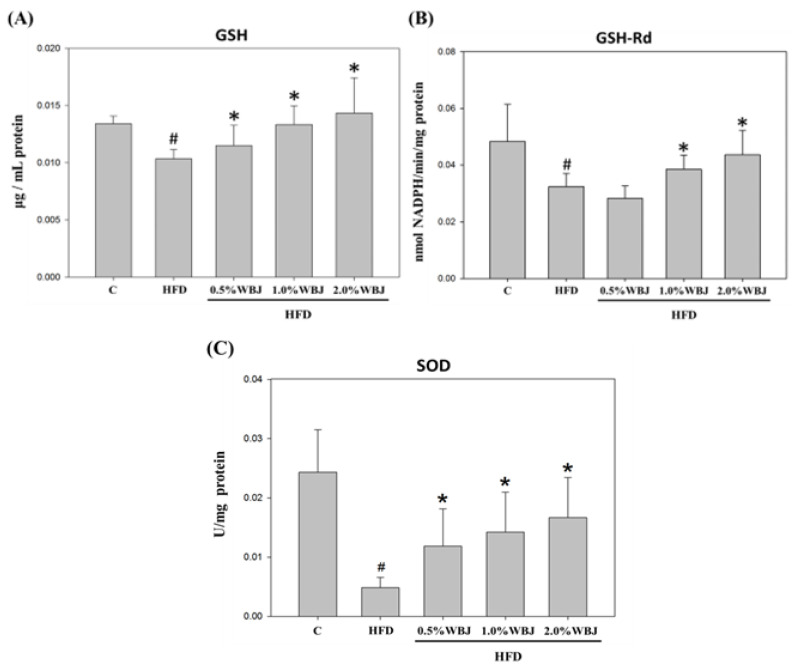
Effects of WBJ on the antioxidant enzyme activities in the liver of HFD-fed rats. The levels of GSH (**A**), GSH-Rd (**B**), and SOD (**C**) in the liver tissue of rats were measured as described in the Materials and Methods. All values are presented as the mean ± SD (n = 12). Results were statistically analyzed with a one-way ANOVA. ^#^ *p* < 0.05 compared to the control group. * *p* < 0.05 compared to the HFD group.

Severe liver lipid accumulation may promote the generation of oxidative stress, leading to the deterioration into NAFLD [24]. We further explored the effect of WBJ on reducing the liver oxidative stress induced by an HFD. After feeding rats with an HFD for 12 weeks, the antioxidant enzyme activities of glutathione (GSH), glutathione reductase (GSH-Rd), and superoxide dismutase (SOD) in the liver showed a significant decrease compared to the control group. However, after treatment with WBJ, the liver antioxidant enzyme activity rose compared to the HFD group in a dose-dependent manner. Therefore, it could be concluded that WBJ can diminish NAFLD induced by an HFD by increasing the activity of antioxidant enzymes in the liver of rats (Figure 6).

### 3.7. The Cytotoxicity Effects of Brassica juncea Glucosinolates on HepG2 Cells

**Figure 7:**
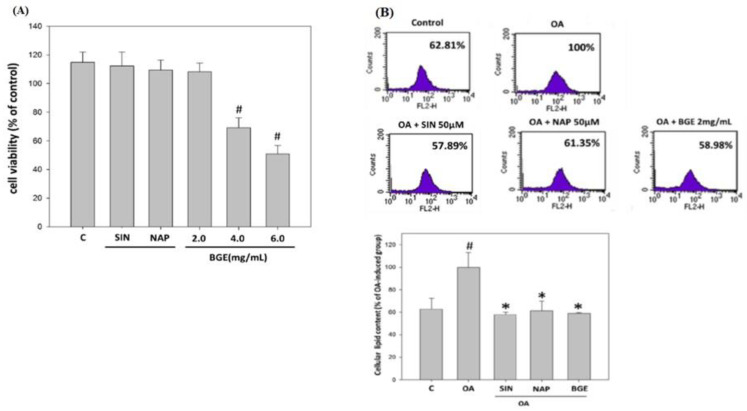
Effects of *Brassica juncea* glucosinolates on lipid accumulation in HepG2 cells. HepG2 cells were incubated with different glucosinolates (50 μM SIN, NAP, and 2.0, 4.0, 6.0 mg/mL BGE) at 37 °C for 24 h. (**A**) Cell viability was analyzed with an MTT assay. (**B**) HepG2 cells were stained with Nile red and analyzed using flow cytometry. The data are presented as the mean ± SD for three replicates per treatment. ^#^ *p* < 0.05 compared to the control group. * *p* < 0.05 compared to the HFD group.

Glucosinolates are a group of phytochemicals that have been shown to be beneficial against a wide range of chronic liver diseases [25]. HepG2 cells were treated with 50 μM SIN, 50 μM NAP, 2.0 mg/mL BGE, 4.0 mg/mL BGE, and 6.0 mg/ml BGE for 24 h, and the cell viability was tested with an MTT assay. The survival rate of cells in the 4.0 mg/mL BGE and 6.0 mg/mL BGE groups showed a significant decrease compared to the control group. Therefore, the dosage that did not significantly cause the death of HepG2 cells (50 μM SIN, NAP, and 2.0 mg/mL BGE) was selected for follow-up experiments (Figure 7A).

### 3.8. Brassica juncea Glucosinolates Inhibited the Lipid Accumulation in HepG2 Cells

To evaluate the effect of glucosinolates on lipid accumulation in hepatocytes, HepG2 cells were exposed to oleic acid (OA); then, 50 μM SIN, 50 μM NAP, and 2.0 mg/mL BGE were added at the same time, for 24 h. The lipids in the cells were stained with Nile red and analyzed quantitatively with flow cytometry. The results showed that the lipid content in HepG2 cells in the SIN, NAP, and BGE groups was lower than that in the induction group, and reducing SIN by about 40% had the most significant effect (Figure 7B). Therefore, SIN and BGE were used in the subsequent experiment.

### 3.9. The Mechanisms of Brassica juncea Glucosinolates Reversing NAFLD

**Figure 8:**
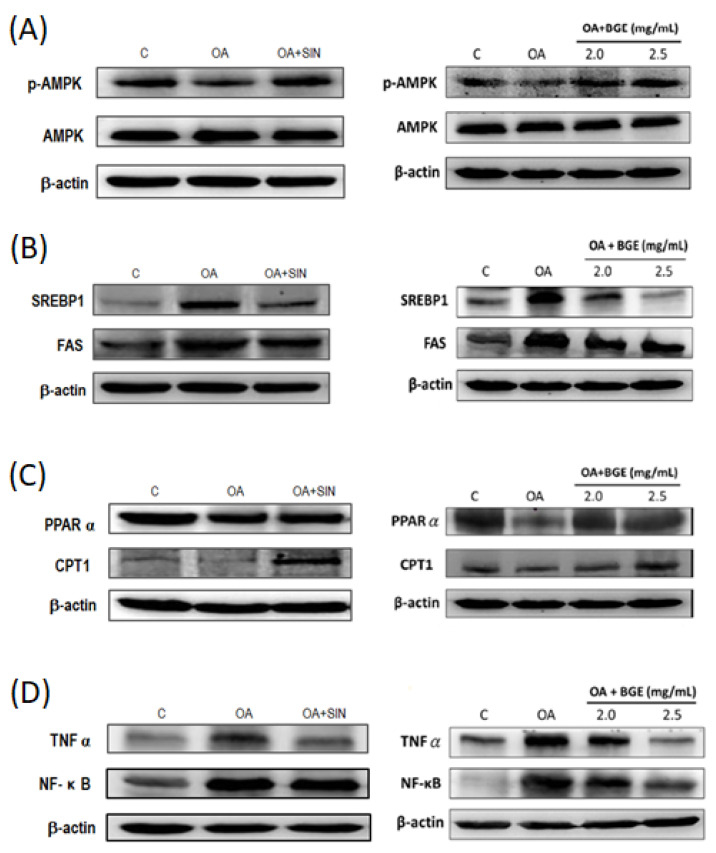
*Brassica juncea* glucosinolates reduced lipid synthesis-related protein and inflammatory protein expression in OA-induced HepG2 cells. HepG2 cells were treated with 0.3 mM OA and 50 μM SIN or different concentrations of BGE (2.0, 2.5 mg/mL BGE) at 37 °C for 24 h. Protein extracts from HepG2 cells were measured with western blotting to detect (**A**) p-AMPK, AMPK; (**B**) SREBP-1, FAS; (**C**) PPARα, CPT1; and (**D**) TNF-α, NF-κb. β-actin was used as a loading control. Data are presented as the mean ± SD from three independent experiments and statistically analyzed with a student’s *t*-test.

Previous studies have demonstrated that the activation of AMPK reduced the expression of triglyceride synthesis proteins, such as SREBP-1 and FAS, activated the expression of fatty acid β-oxidation proteins, such as CPT-1 and PPARα, and then inhibited the occurrence of NAFLD [26]. Our results showed that OA induced excessive lipid accumulation and inhibited the activity of p-AMPK in HepG2 cells, whereas SIN and BGE restored the p-AMPK activity (Figure 8A). Moreover, SIN and BGE decreased the levels of SREBP1 and FAS in HepG2 cells induced by OA (Figure 8B). Meanwhile, SIN and BGE accelerated the lipid β-oxidation via increasing CPT-1 and PPARα expression reduced by OA (Figure 8C). Inflammation plays a crucial role in the progression from NAFLD to more severe non-alcoholic steatohepatitis (NASH) [27]. Figure 8D shows that SIN inhibited TNF-α and NF-κB expression induced by OA in HepG2 cells, whereas BGE inhibited the inflammation of HepG2 cells in a dose-dependent manner. In summary, it can be concluded that *Brassica juncea* glucosinolates SIN and BGE can regulate lipid synthesis and inflammatory factors by activating AMPK, and these glucosinolates can also produce lipid-lowering and anti-inflammatory effects.

## 4. Discussion

The pathogenesis of NAFLD is closely related to obesity, and the World Health Organization (WHO) regards obesity as the most important public health issue in the 21st century. Studies have shown that obesity induces oxidative stress and lipid peroxidation in the body. Lipid accumulation, oxidative stress, and inflammation have been widely regarded as the key factors for simple steatosis, leading to NASH [4,28,29]. Unfortunately, there are still many limitations to the medical treatment of NAFLD induced by a high-fat diet. So far, diet control and exercise are the main suggestions to treat NAFLD because there are currently no FDA-approved medications for this disease. Therefore, if the supplement of natural products can help people prevent or ameliorate NAFLD, it will improve the health quality of the whole society. Our laboratory previously demonstrated that the functional components from *Mulberry* leaf, *Nelumbo nucifera* leaf, and *Hibiscus sabdariffa* can effectively reduce body fat and improve alcoholic and non-alcoholic fatty liver disease [30,31,32]. The hypoglycemic, neuroprotective, antibacterial, and anticancer effects of *Brassica juncea* have been proven previously [33]. Here, our results showed that WBJ reduced body weight, body fat, and hepatomegaly in rats induced by an HFD. In both in vivo and in vitro experiments, WBJ and BGE upregulated p-AMPK expression and exhibited antioxidant enzyme activities, thereby helping to reduce lipid synthesis and promote fatty acid β-oxidation, as well as reducing the production of inflammatory factors.

In the serum lipid parameters, we observed that WBJ had a stronger effect on improving HDL values than reducing LDL values following treatment with WBJ (Table 2). This might be the reason why WBJ decreased the CHO level induced by an HFD in rats in a dose-dependent manner. Moreover, the levels of serum CHO, TG, or FFA, whether given 0.5% WBJ, 1.0% WBJ, or 2.0% WBJ, were significantly lower than those in the HFD group. Therefore, WBJ indeed had a lipid-lowering effect, and it had a significant effect at a low dose (0.5% WBJ) (Table 2).

*Brassica juncea* is rich in dietary fibers (3.2 g dietary fiber/100 g), which can promote gastrointestinal peristalsis, the digestion and absorption of nutrients, and regulate the interactions among intestinal microorganisms [34,35]. In addition, several studies have demonstrated that dietary fiber can elevate the excretion of lipids, reduce the production of cytokines in white adipose tissue, and inhibit systemic inflammation [36,37]. This study confirmed that WBJ promoted lipid excretion in feces. However, during the experiment, we found that there was an occult blood reaction in rat feces from the sixth week after an HFD, which may have been caused by the long-term intake of the HFD [38]. Fecal TG and CHO analyses were therefore limited in the later stage of the experiment. Nevertheless, we still found that WBJ promoted lipid excretion in the third and fifth weeks after the HFD treatment (Figure 3). The imbalance of gut microbiota causes abnormal lipid metabolism in the adipose tissue and liver, as well as immune disorders, which are closely related to metabolic syndromes such as obesity, fatty liver, diabetes, and cardiovascular disease [39,40]. In the future, we will evaluate the effect of functional components of WBJ on increasing beneficial intestinal bacteria and reducing bad bacteria, and we will further explore the role of *Brassica juncea* in disease prevention.

Increasingly, studies have indicated that the AMPK signaling pathway plays a vital role in improving lipid metabolism disorders [41]. LKB1 (liver kinase B1, also known as serine/threonine kinase 11) phosphorylates AMPKα^Thr-172^, thus activating AMPK. Following this, activated AMPK (p-AMPK) inhibits the synthesis pathways of ATP consumption (such as glycogenesis, glycogen synthesis, fatty acid, and cholesterol synthesis) and promotes catabolism (such as fatty acid oxidation, glycolysis) to produce adenosine triphosphate (ATP) [7]. In addition to the LKB1 signaling pathway, there are other AMPK upstream kinases (AMPKK) that can activate AMPK, such as CaMKK and TAK1, to maintain energy balance [26,42]. Our results also showed that WBJ and BGE activated AMPK, inhibited the pathway of SREBP1/FAS, SREBP2/HMGCoR, reduced the synthesis of TG and CHO, and promoted the expression of CPT1 to increase the β-oxidation of fatty acids, thereby reducing the accumulation of lipids in the liver. In the future, the effect of *Brassica juncea* on the expression of LKB1, CaMKK, and TAK1 proteins will be evaluated to further explore the possible mechanism of mustard in improving NAFLD.

Nowadays, for many health foods, it is known that excessive supplementation is not beneficial for health. Indeed, high doses of glucosinolates may have side effects on the body. After feeding rats with 50 μmol/kg sinigrin or its degradation product, allyl isothiocyanate (25 μmol/kg and 50 μmol/kg), for 4 h, rats showed mild hyperglycemia. After continuously feeding sinigrin and allyl isothiocyanate for two weeks, the rats exhibited hyperinsulinemia, hyperlipidemia, and other systemic metabolic disorders [43]. However, the detailed mechanism of metabolic abnormalities caused by excessive glucosinolates is still not fully understood, which is also one of the issues that we need to clarify in the future.

Cruciferae plants are vegetables that are often eaten in our daily life, such as broccoli, cabbage, Chinese cabbage, and mustard. Glucosinolates, a unique component of Cruciferae, have been proved to be a multi-target natural substance that can effectively lower body fat. Glucosinolates also have detoxifying, anti-inflammatory, antioxidant, and anticancer effects [44]. However, rich secondary metabolites, such as glucosinolates, lead to the spicy and bitter taste of some cruciferous plants. The bitterness of cruciferous plants is often associated with consumer rejection and poor taste. If functional components, such as BGE, can be extracted and applied to the development of hepatoprotective nutrient foods, it is believed that its economic value can effectively be improved.

## Figures and Tables

**Table 1:** Feed formulation of Wistar rats.

Ingredients (g/kg Dietary Weight)	Control		HFD		
		-	LD	MD	HD
Casein	260	260	260	260	260
Corn sstarch	500	150	150	150	150
Sucrose	90	90	90	90	90
Corn oil	50				
Beef tallow		400	400	400	400
Cellulose	50	50	50	50	50
Mineral mixture *^a^*	40	40	40	40	40
Vitamin mixture *^a^*	10	10	10	10	10
WBJ			5	10	20

*^a^*, Mineral and vitamin mixtures (AIN-76) were purchased from Oriental Yeast (Tokyo, Japan). Control, normal diet; HFD, high-fat diet; LD, low dose (contained 0.5% WBJ); MD, middle dose (contained 1.0% WBJ); HD, high dose (contained 2.0 % WBJ). WBJ, whole-plant *Brassica juncea*.

**Table 2:** Effects of WBJ on the serum biochemical parameters and hepatic lipids in HFD-fed rats.

	Control	HFD	HFD + 0.5% WBJ	HFD + 1.0% WBJ	HFD + 2.0%WBJ
Total cholesterol (mg/dL)	70.9 ± 3.57 *^a^*	71.3 ± 9.25	51.9 ± 6.19 *^c^*	55.2 ± 7.09 *^c^*	59.2 ± 7.23 *^c^*
Total triglyceride (mg/dL)	34.7 ± 7.24	121.4 ± 26.98 *^b^*	64.4 ± 8.77 *^c^*	74.2 ± 16.47 *^c^*	62.1 ± 15.09 *^c^*
FFA (mmol/L)	0.73 ± 0.10	1.12 ± 0.20 *^b^*	0.77 ± 0.16 *^c^*	0.95 ± 0.07 *^c^*	0.85 ± 0.10 *^c^*
LDL-C (mg/dL)	11.4 ± 3.27	18 ± 3.20 *^b^*	15.8 ± 1.75	13.9 ± 2.13 *^c^*	12 ± 2.11 *^c^*
HDL-C (mg/dL)	49.3 ± 5.96	30.8 ± 4.78 *^b^*	36.1 ± 6.31 *^c^*	37.4 ± 7.72 *^c^*	43.1 ± 6.61 *^c^*
LDL-C/HDL-C ratio	0.23 ± 0.05	0.60 ± 0.15	0.45 ± 0.09 *^c^*	0.38 ± 0.08 *^c^*	0.28 ± 0.06 *^c^*
Glucose (mg/dL)	155.7 ± 52.95	267.7 ± 51.48 *^b^*	235 ± 71.04	268.6 ± 65.61	248 ± 38.34
AST (U/L)	121.7 ± 10.45	140.2 ± 27.32	145 ± 26.44	133.8 ± 17.81	127.9 ± 11.76
ALT (U/L)	33.2 ± 3.91	59.4 ± 9.16 *^b^*	49.3 ± 5.81 *^c^*	44.2 ± 7.45 *^c^*	41.6 ± 8.42 *^c^*
BUN (mg/dL)	20.64 ±1.81	12.92 ± 1.37 *^b^*	9.74 ± 0.92 *^c^*	11.4 ± 0.96 *^c^*	10.54 ± 0.39 *^c^*
UA (mg/dL)	4.01 ± 1.25	4.58 ± 1.05	4.75 ± 1.03	5.13 ± 1.34	4.93 ± 1.22
Creatinine (mg/dL)	0.61 ± 0.05	0.68 ± 0.04 *^b^*	0.62 ± 0.07 *^c^*	0.66 ± 0.05	0.63 ± 0.04
Liver-triglyceride (mg/dL)	253.74 ± 30.21	328.05 ± 26.48 *^b^*	309.23 ± 58.54	265.17 ± 54.82 *^c^*	194.04 ± 58.57 *^c^*
Liver cholesterol (mg/dL)	38.12 ± 12.43	136.38 ± 21.00 *^b^*	113.12 ± 31.21	99.96 ± 27.93 *^c^*	96.69 ± 39.85 *^c^*

*^a^* Each value is expressed as the mean ± SD (n = 10/ group). Results were statistically analyzed with a one-way ANOVA. *^b^ p* < 0.05 compared to the control group. *^c^ p* < 0.05 compared to the HFD group.

**Table 3:** WBJ reduced organ peripheral fat weight in HFD-fed rats.

Tissue Weights (mg)	Control	HFD	HFD + 0.5% WBJ	HFD + 1.0% WBJ	HFD + 2.0% WBJ
Kidney fat	21.63 ± 25.56 *^a^*	7766.00 ± 1408.37 *^b^*	5978.75 ± 1844.83 *^c^*	5622.25 ± 1346.33 *^c^*	5310.25 ± 1976.94 *^c^*
Intestinal fat	331.63 ± 230.70	8306.75 ± 2210.45 *^b^*	6125.63 ± 1394.05 *^c^*	6037.50 ± 994.79 *^c^*	5783.75 ± 577.86 *^c^*
Gonad fat	326.63 ± 116.79	5936.75 ± 874.64 *^b^*	4827.13 ± 1891.91	4766.75 ± 1046.01	4426.00 ± 1184.69 *^c^*

*^a^* Each value is expressed as the mean ± SD (n = 12/group). Results were statistically analyzed with one-way ANOVA. *^b^ p* < 0.05 compared to the control group. *^c^ p* < 0.05 compared to the HFD group.

## Data Availability

No new data were created or analyzed in this study. Data sharing is not applicable to this article.
